# Genome-wide Screen of *Pseudomonas aeruginosa* in *Saccharomyces cerevisiae* Identifies New Virulence Factors

**DOI:** 10.3389/fcimb.2015.00081

**Published:** 2015-11-16

**Authors:** Rafat Zrieq, Thibault G. Sana, Sandra Vergin, Steve Garvis, Irina Volfson, Sophie Bleves, Romé Voulhoux, Johannes H. Hegemann

**Affiliations:** ^1^Institut für Funktionelle Genomforschung der Mikroorganismen, Heinrich-Heine-Universität DüsseldorfDüsseldorf, Germany; ^2^Laboratoire d'Ingénierie des Systèmes Macromoléculaires (UMR7255), Institut de Microbiologie de la Méditerranée, Centre National de la Recherche Scientifique, Aix-Marseille UniversitéMarseille, France

**Keywords:** *Pseudomonas aeruginosa*, genome-wide screening in *Saccharomyces cerevisiae*, *Caenorhabditis elegans* model, virulence factors, *Pseudomonas* effector candidates (Pec)

## Abstract

*Pseudomonas aeruginosa* is a human opportunistic pathogen that causes mortality in cystic fibrosis and immunocompromised patients. While many virulence factors of this pathogen have already been identified, several remain to be discovered. In this respect we set an unprecedented genome-wide screen of a *P. aeruginosa* expression library based on a yeast growth phenotype. Fifty-one candidates were selected in athree-round screening process. The robustness of the screen was validated by the selection of three well known secreted proteins including one demonstrated virulence factor, the protease LepA. Further *in silico* sorting of the 51 candidates highlighted three potential new *Pseudomonas* effector candidates (Pec). By testing the cytotoxicity of wild type *P. aeruginosa* vs. *pec* mutants toward macrophages and the virulence in the *Caenorhabditis elegans* model, we demonstrated that the three selected Pecs are novel virulence factors of *P. aeruginosa*. Additional cellular localization experiments in the host revealed specific localization for Pec1 and Pec2 that could inform about their respective functions.

## Introduction

The bacterium *Pseudomonas aeruginosa* is the principal Gram-negative causative agent of nosocomial infection (Driscoll et al., 2007). It is an opportunistic human pathogen causing acute and chronic infections in immunocompromised individuals. Both invasive and extracellular *P. aeruginosa* strains affect host cellular processes by secreting an important arsenal of effector proteins in the extracellular medium or directly into the host by the means of highly specific secretion machinery (Bleves et al., 2010).

In the last decade, several high throughput screens have been utilized to identify *P. aeruginosa* effectors involved in infection, for instance using: (i) microarrays and RNA-Sequencing to monitor bacterial gene expression during infection of eukaryotic host cells (Wolfgang et al., 2003; Chugani and Greenberg, 2007; Wurtzel et al., 2012; Skurnik et al., 2013), (ii) bacterial mutant libraries to identify virulence-attenuated strains (Feinbaum et al., 2012), (iii) a set of target genes to evaluate their toxicity when produced in yeast (Arnoldo et al., 2008), (iv) high-throughput sequencing of transposon libraries to identify the contribution of individual genes to the fitness of organisms in different environments (Skurnik et al., 2013), (v) mass spectrometry identification of secretomes (Russell et al., 2012), or (vi) bioinformatic approaches (Jehl et al., 2011; Burstein et al., 2015). However, despite the high throughput of these approaches, new *P. aeruginosa* effectors are still regularly revealed (Sana et al., 2012; Russell et al., 2013; Faure et al., 2014; Burstein et al., 2015) and many others certainly remain to be discovered. Their identification will improve the understanding of *P. aeruginosa* infection leading to the development of new alternative therapeutic strategies.

There are increasing evidences for the utility of the yeast *Saccharomyces cerevisiae* model to discover new bacterial effector proteins of human pathogens. This relies on the observation that the bacterial effector proteins often target cellular processes that are conserved among eukaryotes, from yeast to human. Thus, expression of bacterial effector genes in yeast alters yeast pathways and results in a yeast growth defect, for example, *Pseudomonas aeruginosa* ExoU and ExoS (Rabin and Hauser, 2003; Stirling and Evans, 2006)*, Shigella flexneri* IpgB1 and IpgB2 (Slagowski et al., 2008)*, Salmonella typhimurium* SipA and SigD (Lesser and Miller, 2001; Aleman et al., 2005)*, Chlamydia trachomatis* Lda3 (Sisko et al., 2006)*, Chlamydia pneumoniae* CopN (Huang et al., 2008), enteropathogenic *E. coli* EspD and EspG (Rodriguez-Escudero et al., 2005). Interestingly, the subcellular localization of many ectopically produced bacterial effector proteins in yeast mimics the localization of the protein in the host and gives an indication of their possible functions. For example, the *Salmonella* SipA was first identified to localize the yeast actin cytoskeleton and disrupts its polarity by preventing turnover of actin cables (Lesser and Miller, 2001). Further studies in mammalian systems revealed that SipA bundles actin filaments and inhibits their depolymerization (Galkin et al., 2002). Other advantages have indeed been accounted for yeast as a valuable tool to analyse bacterial effector proteins including (i) the ease of cloning by homologous recombination and transformation, (ii) regulation of the expression of target genes, (iii) the availability of yeast mutant strains as well as (iv) the yeast reporter strains for localization studies, (v) the ease of isolating single colonies from DNA library-transformed yeast cells and the (vi) availability of redundant phenotypes of yeast that are defective in certain pathways.

Despite the increasing number of studies characterizing known bacterial effectors in yeast, these approaches were only used to study selected candidates. In this work, we perform for the first time an unbiased genome-wide screen of *P. aeruginosa* PA14 strain to identify potential effector proteins that alter yeast cellular processes and impair yeast growth. By expressing in yeast a genomic library of *P. aeruginosa* PA14, we identify a set of 51 putative effector proteins and validated 3 of them that have never been described so far. This successful study represents the first genome-wide screen of a complete bacterial genome to identify bacterial effector proteins in yeast.

## Materials and methods

### Bacterial strains, eukaryotic strains, cell lines, and media

For cytotoxicity and *C. elegans* virulence assays we used the wild type *Escherichia coli* OP50 (laboratory collection) strain, the wild type *P. aeruginosa* PA14 (Liberati et al., 2006) and PA14Δ*gacA* (Garvis et al., 2009) strains as well as the four PA14 mutants, 23531, 30063, 54050, and 41532 from the PA14 Transposon Insertion Mutant Library (http://ausubellab.mgh.harvard.edu/cgi-bin/pa14/home.cgi) which respectively possess a transposon inserted in *pec1 (PA14::tn-pec1), pec2 (PA14::tn-pec2), pec3 (PA14::tn-pec3)*, and *exoU (PA14::tn-exoU)*. The *Saccharomyces cerevisiae* strains used in this study are the wild-type CEN.PK2 (EUROSCARF) and the mRFP reporter strains (mRFP-Pex3 and mRFP-Erg3) for colocalization studies (Huh et al., 2003). Plasmid-bearing strains were grown on selective synthetic dextrose (SD) medium supplemented with 2% glucose, raffinose or galactose as desired. HEp-2 cells (ATCC: # CCL-23) were routinely cultured in DMEM medium (Invitrogen) supplemented with 10% fetal calf serum (FCS; Invitrogen).

### DNA manipulation, library, and plasmid constructions

pSR1 (*HIS3 GAL1*-*His*_6_ 2 μ), a p423GAL1-derived plasmid (Mumberg et al., 1994) bearing a PCR-amplified cassette (Start codon-linker-his6 tag-3 stop codons for 3 frames) into the EcoRV site, was used for constructing the *P. aeruginosa* PA14 genomic library. The library used in this study was constructed (by AGWOA GmbH, Germany) as follows: *P. aeruginosa* PA14 genomic DNA was prepared following the protocol in Current Protocols in Molecular Biology, 1997 (Unit 2.4) (Wilson, 2001). The obtained pelleted DNA was then resuspended in TE and then fragmented by sonication into 500–1000 bp sections and inserted into *EcoR*V site of the library empty vector pSR1 (constructed by AGWOA GmbH, Germany). Construction of plasmids used in cellular localization experiments are described in the supplementary text.

To generate *pec2* deletion, 500 bp upstream, and 500 bp downstream of the *pec2* gene (*PA14_03100*) were amplified by overlapping PCR with High Fidelity DNA polymerase (Roche Applied Science) using primers TSO87(5′ACCCTGCTCCTCTGCCTTTGCC3′), TSO88(5′C TA ATGCAGTATCCGGTTCATCTTCAGTCCTCGGAGTGG3′,TS O89 (5′GGACTGAAGATGAACCGGATACTGCATTAGGTCG CCG3′ and TSO90 (5′ACTGGCCGTCTT CCACGACCG3′). The PCR product was cloned in pCR2.1 (TA cloning kit; Invitrogen) giving pTS39, which was then sequenced (GATC) and sub-cloned in pKNG101 suicide vector (Kaniga et al., 1991) giving the mutator pTS41. pTS41, maintained in the *E. coli CC118*λ*pir* strain, was mobilized in the wild type *P. aeruginosa* strain PA14. The mutants, in which the double recombination events occurred that resulted in the nonpolar deletion of *pec2* gene (*PA14_03100*), were verified by PCR using external primers TSO99 (5′GCCCTGGGCAAGTTGCTGCG3′) and TSO100 (5′GAGGGTGGCATCGCGGTCGG3′). Full length genes of *P. aeruginosa* PA14 was cloned in yeast and human expression vectors are described in details in Supplementary Information.

### Screening of *P. aeruginosa* genomic library in yeast

1.14 μg of the library (representing 3 times coverage of the PA14 genome) was transformed into yeast and platted onto non-inducing media agar plates. The screen was then processed in 3 steps. First, the expression of the transformed fragments in obtained yeast transformants was induced by transferring yeast colonies via replica platting onto media agar plates containing 2% of the inducer galactose. To assess the impact of expression of library inserts on yeast growth, Phloxin B that stains dead cells in pink was added to the media. Colonies on inducing media were compared to their replicates on non-inducing media and those that did not grow, displayed smaller size and/or increased pink staining on inducing media plates were selected. These initial candidates were then subjected to a secondary screen by the serial dilution patch test. In this assay, a series of yeast cell dilutions was dropped on non-inducing and inducing media and the growth of yeast cells, represented by the number and size of colonies in comparison to control cells, was assessed. Yeast candidates showing a growth arrest phenotype were selected. Plasmid DNA was then isolated from each yeast colony and transferred into competent *E.coli* cells. Plasmids from *E.coli* strains (from at least 3 *E.coli* transformants for each yeast candidate) were isolated and restriction analysis performed to identify those yeast candidates harboring plasmids carrying different inserts. Individual plasmids were then transformed into yeast and subjected to a tertiary screen consisting in a serial dilution patch test. Plasmid inserts from yeast candidates showing a growth phenotype were isolated and their *P. aeruginosa* PA14 genomic fragment were sequenced and corresponding genes were subjected to further analysis.

### Serial dilution patch test

Patch tests were performed with dilution series prepared from yeast cultures in exponential phase (OD_600_: ~ 0.9) grown in SD-HIS supplemented with 2% raffinose. Dilutions were then spotted on either selective agar medium (SD-HIS) supplemented with 2% raffinose or galactose and the plates were incubated at 30°C for 48 h.

### Transfection and staining of HEp-2 cells

HEp-2 cells grown overnight on coverslips were transfected with 1 μg of the desired DNA plasmid using Turbofect (Thermo Scientific) as recommended by the manufacturer. After the desired time (see Figure legends) at 37°C with 5% CO_2_, cells were washed with 1x PBS, fixed in 3.7% paraformaldehyde. For staining of microtubule, γ-tubulins or peroxisomes, cells were permeabilized with 0.1% Triton X-100, incubated with a monoclonal mouse anti-alpha tubulin (Acris), a monoclonal mouse anti-γ-tubulins (Sigma-Aldrich) or a monoclonal mouse anti-PMP70 primary antibodies (Sigma-Aldrich), and then with proper labeled secondary antibodies. To stain lipid droplets, fixed HEp-2 cells were permeabilized with 0.5% saponin and stained with Bodipy 493/503 (Molecular Probes). Actin staining of human cells with rhodamine-phalloidin was performed as described by the manufacturer (Molecular Probes). DNA was stained with Dapi. Microscopy was carried out using either spinning-disk confocal microscopes (Carl Zeiss).

### Cytotoxicity assay

The cytotoxicity of the parental PA14 strain and, its isogenic *pec1-3* insertional mutants were assayed by using murine RAW 264.7. RAW macrophages were routinely grown in 96-well plates in Dulbecco modified Eagle medium, GlutaMAX, sodium pyruvate, and phenol red supplemented with 10% FBS. Prior to infection, confluent RAW cells were washed twice with sterile PBS and incubated in RPMI 1640 medium without phenol red. The wild-type *P. aeruginosa* and the *pec* insertional mutants were grown overnight in TSB and sub cultured into fresh TSB to early stationary growth phase, washed with sterile PBS twice, and resuspended in RPMI 1640 medium devoid of FBS. Macrophages cells were infected with bacteria at a Multiplicity of Infection (MOI) of 20 for 45 min at 37°C in 5% CO_2_ incubator. After a centrifugation at 190 X g to sediment the infected cells, 100 μL of culture supernatants were collected and transferred in a new 96-well plate. The release of the cytosolic LDH into the supernatant was measured using a Roche LDH kit in accordance with the manufacturer′s instructions. Percentage of LDH release was calculated relative to that of the uninfected control, which was set at 0% LDH release, and that of lysed uninfected cells, which was set at 100% LDH release. For statistics, a Dunnett′s test has been performed as part of One-Way ANOVA using GraphPad Prism.

### *C. elegans* virulence assay

The slow killing assay was performed as described previously with modifications (Zaborin et al., 2009). Each independent assay consisted of three replicates. *E. coli* OP50 was used as a control. L4 to adult stage *C. elegans* were removed from food and placed on unseeded NGM plates for 24 h at 25°C. 50 worms were then picked onto plates containing overnight growth of each bacterial strain. The worms were evaluated for viability on a daily basis. The assay was performed three times. Animal survival was plotted using the PRISM 5.0 program. Survival curves were considered significantly different from the control when *p*-values were <0.05. PRISM calculated survival fractions using the product limit (Kaplan-Meier) method and compared survival curves by two methods: the log-rank test (also called the Mantel-Cox test) and the Gehan-Breslow-Wilcoxon test. When necessary Gentamicin was used at 15 μg/ml.

## Results

### *P. aeruginosa* PA14 genome-wide screen in yeast identifies genes that alter yeast growth

**Figure 1:**
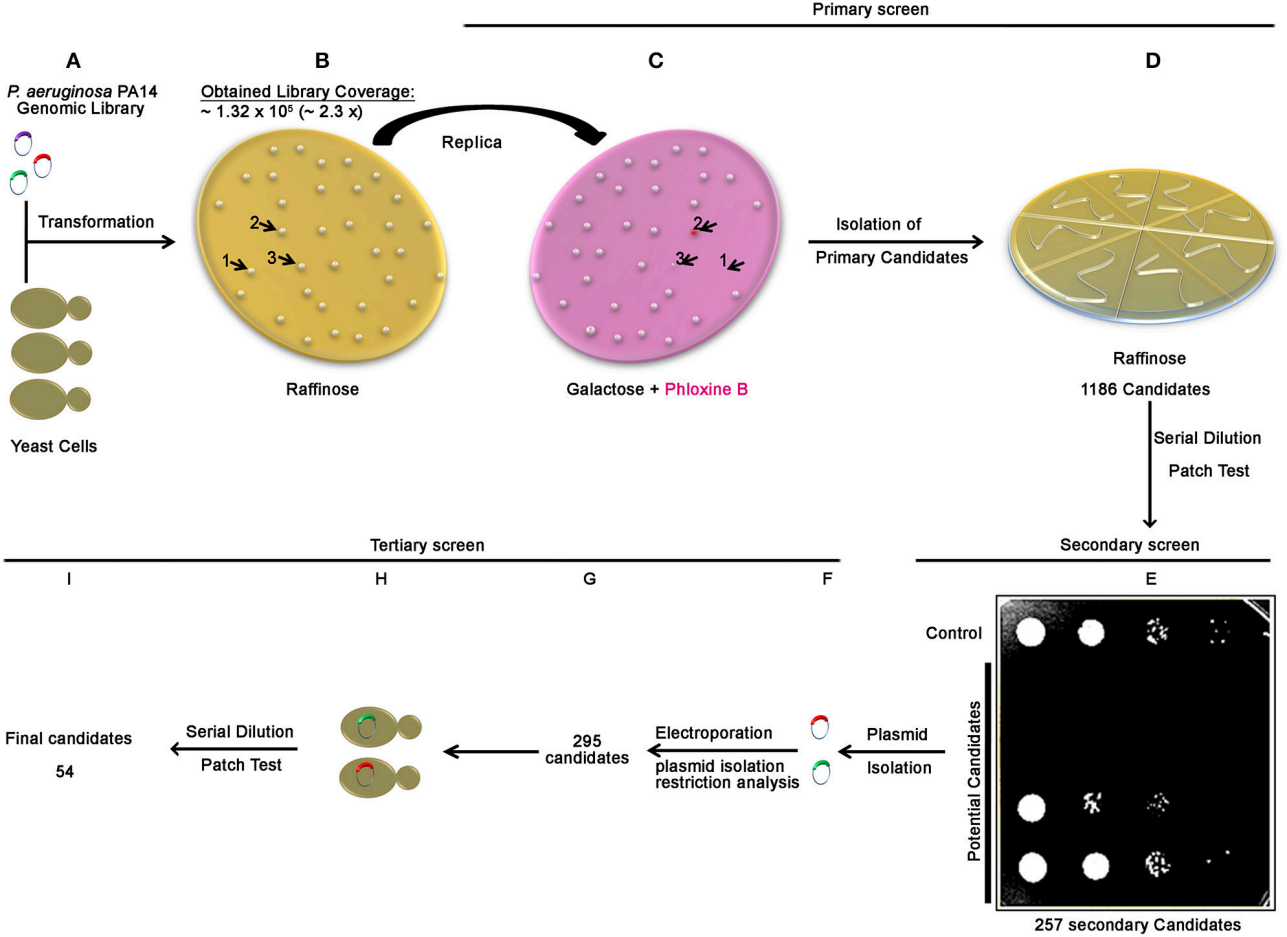
**Scheme of the genome wide screen of ***P. aeruginosa*** PA14 in yeast**. The correlation between amount of library DNA and genome fold was determined by transforming yeast cells with serial dilutions of the library DNA and counting the resulting yeast transformants. Having the correlation established, 1.14 μg of the library DNA (representing 3 times coverage of the PA14 genome) was transformed into yeast cells **(A)**. For the primary screen, transformed yeast cells were selected on a synthetic defined medium lacking histidine (SD-HIS) supplemented with raffinose **(B)**. Yeast colonies obtained ~1.3 105 transformants, representing approximately 2.3 times of the PA14 genome) were transferred by replica platting to a solid SD-HIS containing galactose and Phloxine B medium **(C)**, arrows and numbers (1, 2, and 3) in **(C)** represent examples for growth phenotypes observed (non-growing, dead phloxine-positive and smaller colonies, respectively), see also Figure S1. Colonies showed growth phenotype (1186 candidates) were streaked out on plates (SD-HIS) supplemented with raffinose **(D)**. For the secondary screen, independent candidates from **(D)** were subjected to serial dilution patch test and positive Pseudomonas effector candidates were selected **(E)**. Plasmids were then isolated **(F)** for the tertiary screen and differentiated by restriction analysis **(G)**. Plasmids were retransformed into yeast **(H)** and transformants from each candidate were subjected for serial dilution patch test. Fifty four candidates showed growth phenotype **(I)**.

To identify bacterial proteins that perturb essential and conserved eukaryotic processes, we constructed a genomic library of the *P.aeruginosa* PA14 strain and performed a three steps genome-wide screen in yeast for bacterial genomic fragments inducing a yeast growth phenotype. After transforming the library into yeast and platted onto non-inducing media agar plates (Figure 1A), approximately 1.3 × 10^5^ yeast colonies were obtained indicating that at least the equivalent of 2.3**-**fold of the *P. aeruginosa* genome was actually transformed (Figure 1B). The screen was then processed in 3 steps. In the first step (Figures 1C,D), we performed a primary negative selection by transferring yeast colonies via replica platting onto media agar plates containing galactose and Phloxin B (Figure 1C). We identified and isolated 1186 colonies that did not grow, displayed smaller size and/or increased pink staining on inducing media plates (Figures 1C,D and Figure S1). In the secondary screen, performed by a serial dilution patch test, we selected among the 1186 primary candidates, 257 secondary candidates showing a growth arrest phenotype (Figure 1E). Restriction analysis of plasmid DNA from the 257 yeast candidates revealed that 38 yeast candidates harbored two different plasmids that carried two different inserts, resulting in a total of 295 plasmids (Figures 1F,G). Finally, in the tertiary screen, among the 295 plasmids retransformed in yeast (Figure 1H), 54 potential *P. aeruginosa* PA14 genomic fragment carrying-plasmids showed a growth phenotype defect by serial dilution patch test (Figure 1I). All plasmid inserts were sequenced and corresponding genes were subjected to further analysis.

### Evaluation of the screen and *in silico* analysis for selection of potential candidates

**Figure 2:**
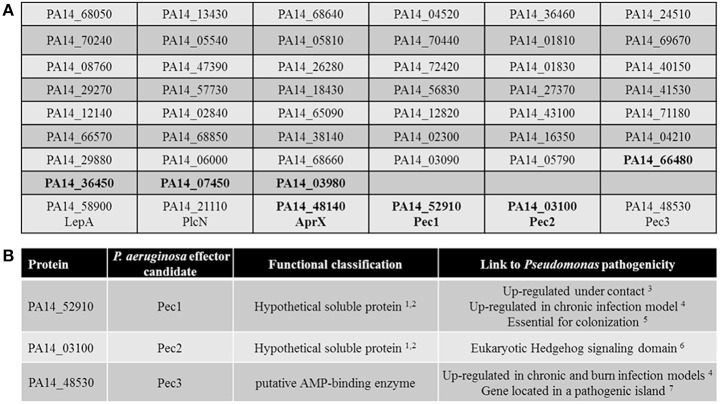
*****In silico*** sorting of the 51 selected candidates**. **(A)** List of the 51 selected candidates. Among the hypothetical proteins (in bold), the soluble ones are in bold. The three known secreted proteins of *P. aeruginosa* as well as the three selected Pecs proteins are indicated by their names. **(B)** Summary of the characteristics of the 3 selected Pecs. ^1^TopPred 0.01 (von Heijne, 1992); ^2^Phobius (Kall et al., 2004) and; ^3^(Chugani and Greenberg, 2007); ^4^(Turner et al., 2014); ^5^(Skurnik et al., 2013); ^6^PFAM (Taylor et al., 2002); ^7^IslandViewer (Langille and Brinkman, 2009).

The genes covered by the sequence of the 54 *P. aeruginosa* PA14 inserts were identified using BLAST. Sequence analysis revealed that three different regions of the genome were found twice in the screen. Further *in silico* analysis revealed that six inserts harbored sequences from two neighboring ORFs, while six other inserts carried ORFs in the wrong orientation or only non-coding sequences and therefore were excluded from further analysis. We thus revealed 51 unique PA14 ORFs inducing a growth phenotype in yeast (Figure 2A and Table S1).

Interestingly, 3 out of the 51 candidates have already been experimentally demonstrated to be secreted exoproteins of *P. aeruginosa* (Figure 2A). The non-hemolytic phospholipase C PlcN (PA14_21110), secreted by the Type II secretion system (T2SS) (Voulhoux et al., 2001), the Type I secretion system (T1SS) effector of unknown function AprX (PA14_48140) (Duong et al., 2001), and the protease LepA (PA14_58900), secreted by a Type V secretion system (T5SS) and contributing to the virulence of *P. aeruginosa* toward human bronchiole epithelial cells (Kida et al., 2008).

In order to discriminate the cytotoxicity mediated by true virulence factors from the one due to the ectopic expression of gene fragments that results in nonspecific growth defect, the 51 gene candidates previously selected were *in silico* screened for features that could classified them as potential new virulence factors such as secreted proteins of unknown function linked to *Pseudomonas* pathogenicity. We thus selected among the 26 hypothetical proteins (*Pseudomonas* genome database website, Pseudomonas.com) (Table S1) the most likely secreted candidates. To do so, we performed further *in silico* analysis based on the criteria that secreted factors are mainly soluble proteins. Using Phobius (Kall et al., 2004) and TopPred 0.01 (von Heijne, 1992) programs, 7 soluble proteins of unknown function were predicted (in bold, Figure 2A), PA14_66480, PA14_36450, PA14_07450, PA14_03980, PA14_48140 (AprX) PA14_52910 and PA14_03100. Among them, the corresponding gene encoding PA14_52910 has been shown to be up regulated under contact with cultured primary differentiated human airway epithelia (Chugani and Greenberg, 2007) as well as in the murine chronic wound infection model (Turner et al., 2014). Moreover, a recent study revealed that this protein is also essential for colonization of the murine gastrointestinal track by *P. aeruginosa* (Skurnik et al., 2013). Thus, candidate PA14_52910 constitutes a putative new secreted virulence factor and therefore was termed Pec1 (*P. aeruginosa* PA14 effector candidate 1, Figure 2B). In addition, the predicted secreted protein of unknown function PA14_03100 has a putative eukaryotic-like domain (Suppressors of fused protein, SUFU) known to repress the Hedgehog signaling pathway, a key regulator of animal development (Taylor et al., 2002), thus constituting a clue to also consider it as a strong effector candidate (Pec2, Figure 2B).

We also searched using the program IslandViewer (http://www.pathogenomics.sfu.ca/islandviewer/query.php (Langille and Brinkman, 2009) if any of the 51 ORFs located within a known or predicted genomic pathogenicity island. Our search revealed that ORF PA14_48530 is found within such a predicted island. Moreover, the corresponding gene has been shown to be up regulated in the murine chronic and burn wound infection models (Turner et al., 2014). Finally and interestingly, this protein (Pec3, Figure 2B) which is a putative AMP-binding protein, is also predicted to be soluble making it an interesting effector candidate as well.

### Expression of full length *pec* genes in yeast induces growth defect phenotype

**Figure 3:**
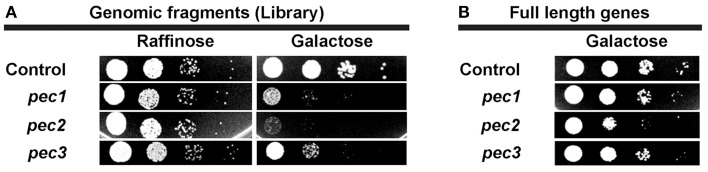
**Yeast growth phenotype induced by expression of selected ***pec*** genes in yeast. (A)** Serial dilution patch test of yeast cells carrying the empty vector (control) or the 3 selected candidates obtained from the screen (Pec1, Pec2, and Pec3). Cells were dropped on selective non-inducing medium (Glucose) or selective induced medium (Galactose) as indicated. **(B)** Serial dilution patch test of yeast cells carrying the empty plasmid (control) or full length genes of the 3 selected candidates (Pec1, Pec2, and Pec3). Cells were dropped on selective induced medium (Galactose) as indicated.

Expression of *pec1,pec2*, and *pec3* from the original screen plasmids resulted in impaired yeast growth (Figure 3A). In order to test whether the expression of full length *pec* genes also would impair yeast growth, we produced the full length Pec1, Pec2, and Pec3 proteins in yeast and tested them by serial dilution patch tests (Figure 3B). In comparison to the yeast growth phenotypes induced by the initial screen plasmids, the production of full length Pecs showed a significant but attenuated yeast growth defect phenotype, as detectable by smaller and less numerous colonies. These differences in growth are likely due to alterations in protein production levels, protein folding and/or protein modification. We checked by sequencing that the differences in growth of yeast cells expressing full length and genomic fragment of Pec1 and Pec2 were not due to enhanced translation given by additional genes or small RNAs within the cloned fragments (data not shown). We therefore concluded that expression of the 3 full-length *pec* genes validated the original yeast growth phenotype, supporting their possible role as *P. aeruginosa* virulence factors.

### Involvement of Pec1 and Pec3 in *P. aeruginosa* cytotoxicity toward macrophages

**Figure 4:**
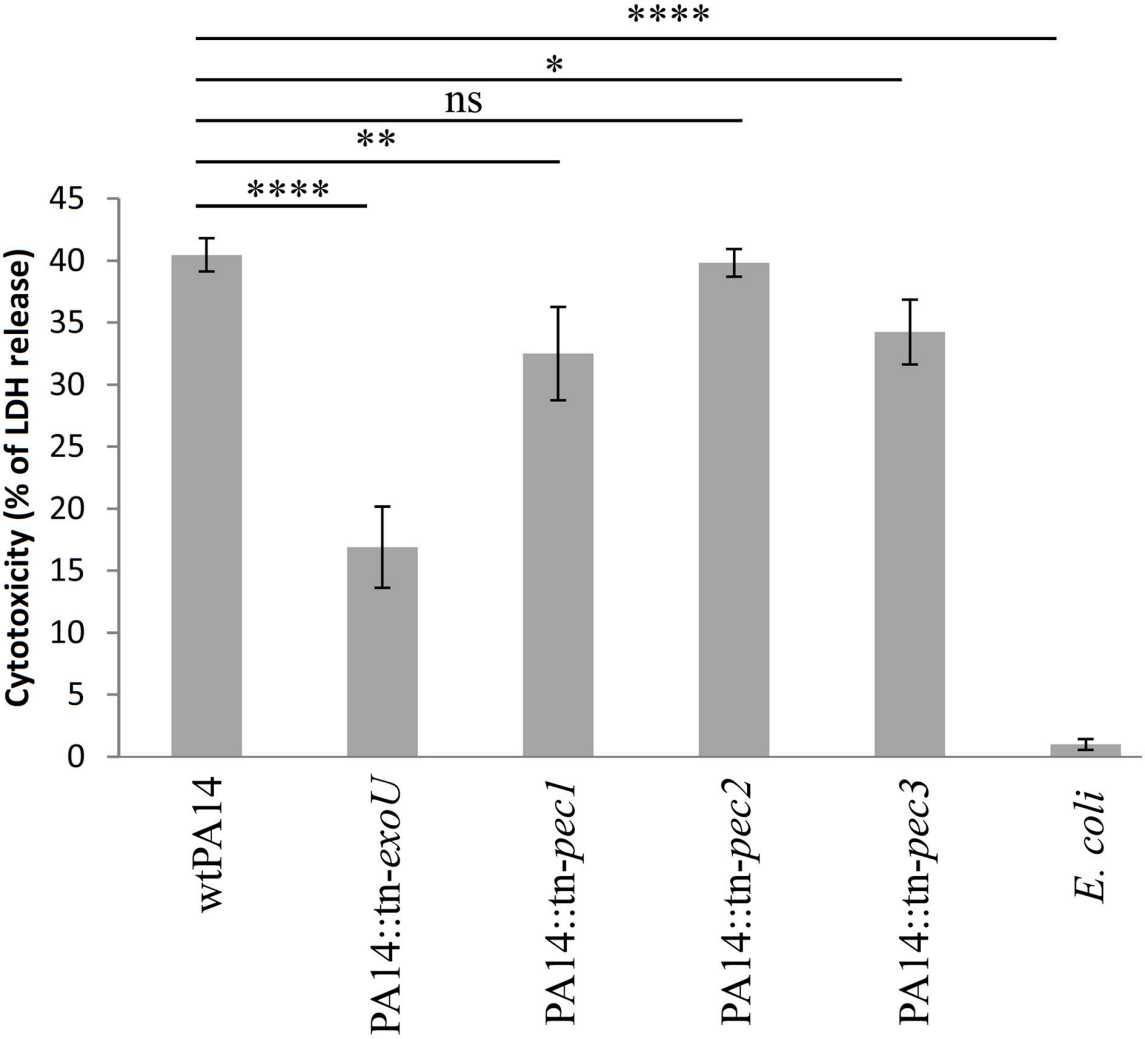
**Involvement of Pec1 and Pec3 in cytotoxicity against macrophages**. RAW264.7 macrophages were infected with the parental *P. aeruginosa* PA14 strain (wtPA14), the negative controls lacking *exoU* gene (PA14*::tn-exoU*) and a non-toxic *E. coli* strain (TG1), the PA14 strains bearing a transposon in *pec1* (PA14::tn-*pec1*), *pec2* (PA14*::tn-pec2*), or *pec3* (PA14*::tn-pec3*) at an MOI of 20 during a 45 min. Cytotoxicity was measured with LDH release assay. Percentage of cytotoxicity is shown on the vertical axis. Error bars show standard deviation. Data shown are representative of four independent experiments. ^****^ for *P* < 0.0001; ^**^ for *P* < 0.01; ^*^ for *P* < 0.1; ns for non-significant.

We further assessed the role of the Pecs on a crucial aspect of host pathogen interaction, the killing of macrophages by *P. aeruginosa*. In this respect, we compared the cytotoxicity toward RAW264.7 macrophages of the wild type PA14 strain with 3 isogenic *pec* mutants. The release of cytosolic lactate dehydrogenase (LDH) was measured after 45 min of macrophage infection at a MOI (Multiplicity of Infection) of 20. The *pec* mutant strains used in this assay belong to the PA14 transposon mutant library generated by Liberati et al. (2006). A significant reduction in cytotoxicity was observed in strains with transposon insertions in *pec1* (PA14 *tn-pec1*) and *pec3* (PA14 *tn-pec3*) while the transposon insertion in *pec2* (PA14 *tn-pec2*) displayed a wild type phenotype (Figure 4). As a positive control, we used the transposon mutant of the library carrying an insertion in *exoU*, the gene encoding the potent cytotoxin ExoU secreted by the Type III secretion system (T3SS) (Hauser, 2009). This mutant presents a strong defect in cytotoxicity against macrophages, thus validating our assay as well as the transposon library. It is moreover not surprising that, although statistically significant, the effects of the Pecs found toward macrophages are relatively weak since most of the *P. aeruginosa* PA14 cytotoxicity is due to ExoU (Figure 4). Through this result we showed that, in agreement with their toxicity in yeast, the selected candidates Pec1 and Pec3 are novel *P. aeruginosa* effectors involved in the cytotoxicity toward macrophages.

### The 3 selected Pecs contribute to *P. aeruginosa* virulence in the *caenorhabditis elegans* worm model

**Figure 5:**
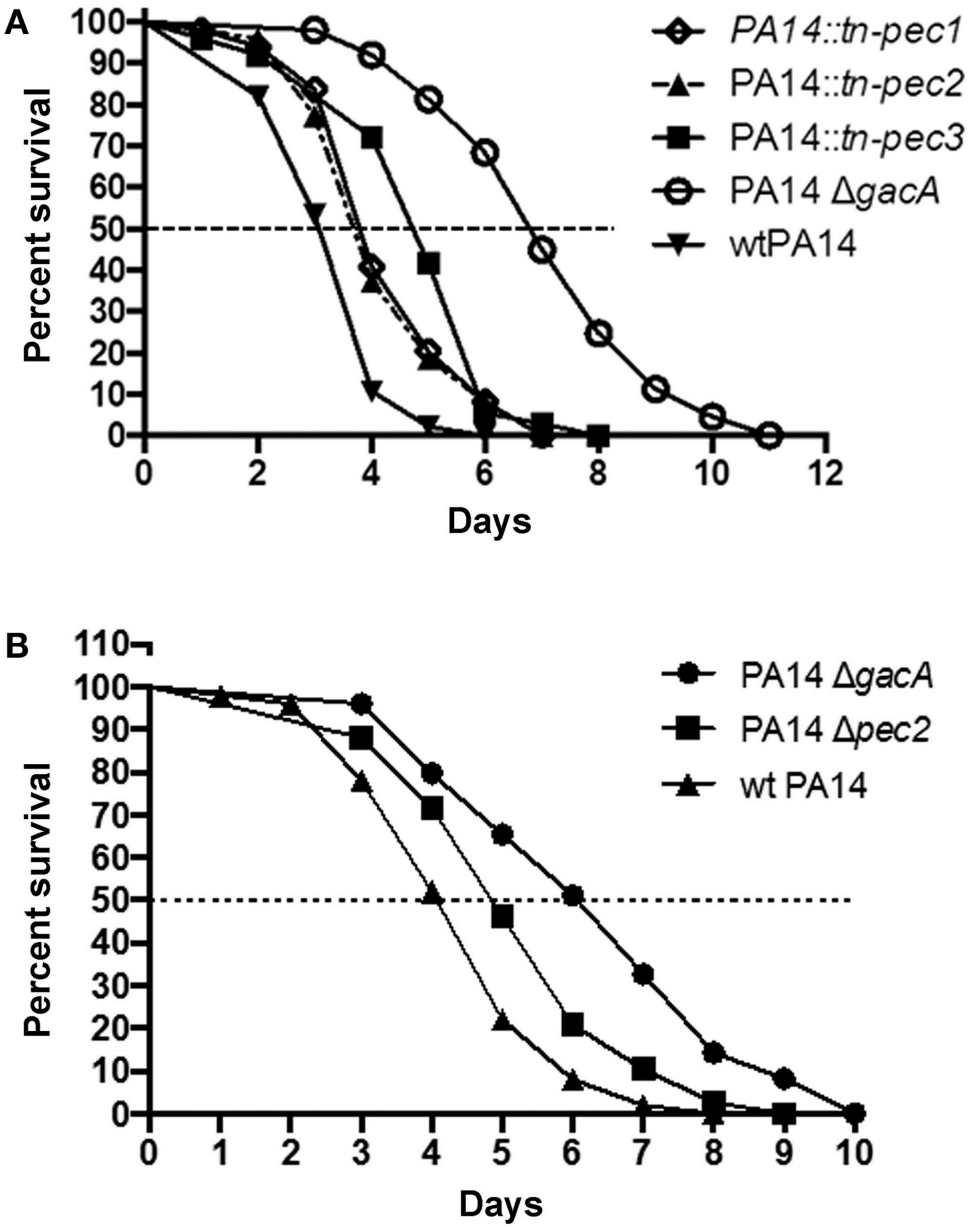
**Involvement of Pec1, Pec2, and Pec3 in PA14 virulence in the ***C. elegans*** model**. Impairment of *pec1, pec2*, and *pec3* decreases the ability of *P. aeruginosa* to kill *C. elegans* in a slow killing assay. Worms were infected with the positive control constituted by the parental *P. aeruginosa* PA14 strain (wtPA14), the negative control lacking *gacA* (PA14Δ*gacA*) and the PA14 strains bearing a transposon in *pec1* (PA14*::tn-pec1*), *pec2* (PA14*::tn-pec2*), or in *pec3* (PA14*::tn-pec3*) **(A)** or lacking *pec2* (PA14Δ*pec2*) **(B)**. The percentage of surviving nematodes is shown with respect to the number of days post-infection. Data presented are representative of at least two independent experiments. See text for *P*-values.

The involvement of Pecs in *P. aeruginosa* pathogenicity led us to test their role in virulence in the *C. elegans* model. Previous studies have shown that *C. elegans* is a good model to identify *P. aeruginosa* virulence factors important for mammalian virulence (Garvis et al., 2009; Feinbaum et al., 2012; Sana et al., 2012, 2013). In a slow killing assay, we observed, in agreement with the cytotoxicity assays, that strains PA14::*tn-pec1* and PA14::*tn-pec3* were significantly less virulent than the PA14 wild type strain [*P* < 0.001 (^***^)] (Figure 5A) thus confirming that Pec1 and Pec3 are virulence factors. Interestingly, Pec2 also contributes to *P. aeruginosa* virulence in this model [*P* < 0.001 (^***^)] revealing that Pec2 is also a virulence factor, although harmless against macrophages. The parental wild type PA14 *P. aeruginosa* strain was used as positive control while the non-virulent PA14 *P. aeruginosa* strain (PA14Δ*gacA*) was used as negative control.

In order to exclude any polar effect on downstream genes due to the transposon insertion in the *pec* gene, we constructed an in frame *pec2* deletion mutant (PA14 Δ*pec2*). The attenuated virulence observed with PA14::*tn-pec2* strain was confirmed with the *pec2* full deletion mutant [*P* < 0.01 (^**^)] (Figure 5B). However, since we were unable to generate in frame deletions of *pec1* and *pec3*, we cannot exclude any polar effect in these cases. It is however unlikely that the reduction of cytotoxicity and virulence observed in PA14::*tn-pec1 and* PA14::*tn-pec3* is not due to *pec1* and *pec3* gene products since they are toxic when expressed in yeast. All together we have shown by two different and independent approaches that the 3 effectors selected in our yeast screen are true new *P. aeruginosa* virulence factors that contribute to the *P. aeruginosa* cytotoxicity against macrophages and/or virulence on the *C. elegans* model.

### Subcellular localization studies of Pecs after expression in yeast and human cells

In order to validate that Pecs have subcellular targets in host cells, we cloned the 3 *pec* genes required for *P. aeruginosa* PA14 infectivity in human expression vectors and transfected them into HEp-2 cells for subsequent analysis by spinning disk microscopy.

#### GFP-Pec3 diffuses in HEp-2 transfected cells

**Figure 6:**
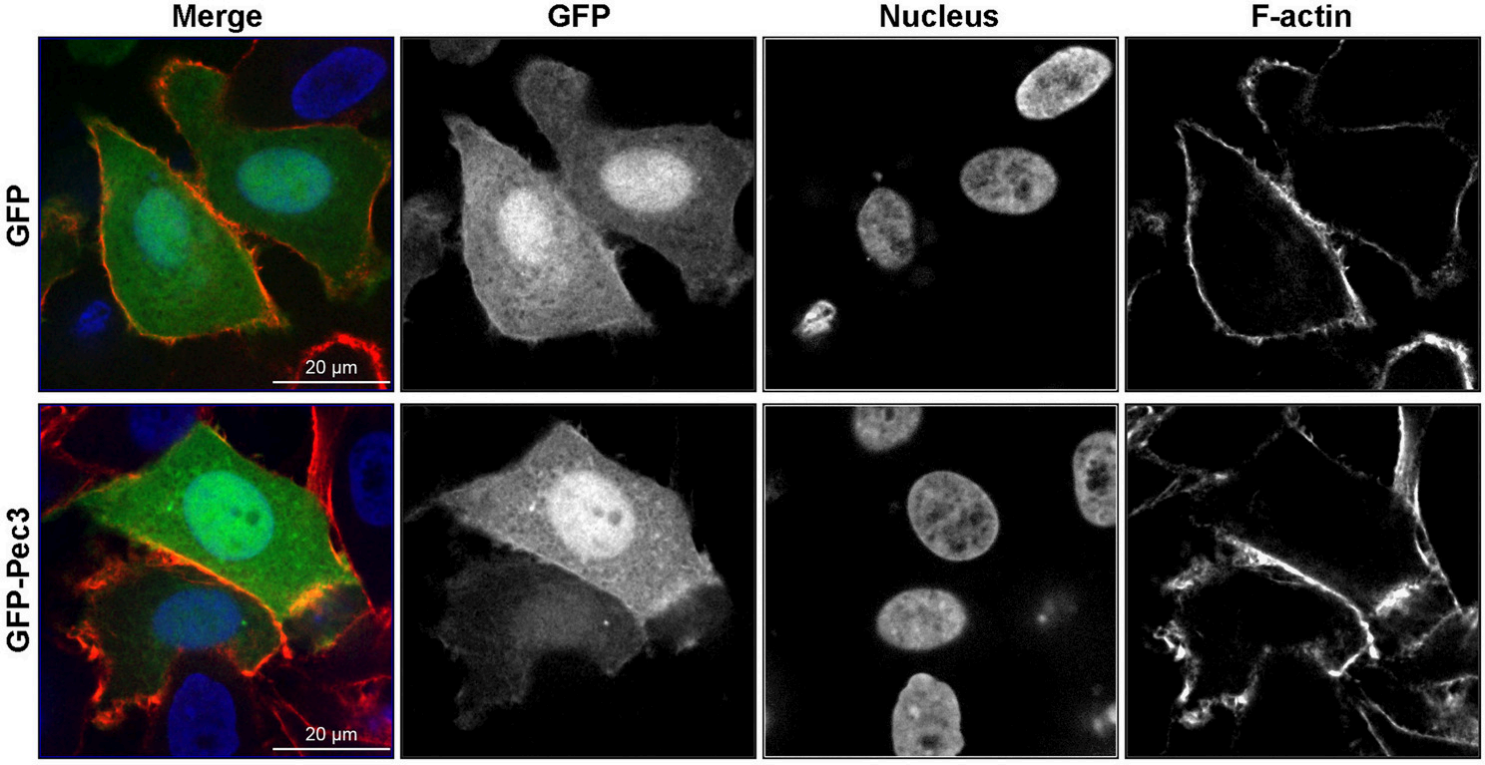
**Expression patterns of GFP-Pec3 in HEp-2 transfected cells**. HEp-2 cells were transfected with a human expression vector carrying GFP or GFP-Pec3 for 18 h. Cells were then fixed and stained with DAPI (blue) to visualize nuclei and with rhodamine-phalloidin (red) to visualize F-actin.

Ectopic production of GFP-Pec3 in epithelial cells showed a diffuse GFP pattern similar to GFP expressing control cells indicating no particular localization of this effector (Figure 6). A similar localization pattern was also observed in yeast (data not shown). These results suggest that the target of Pec3 cannot be identified by localization studies.

#### YFP-Pec1 localizes to microtubule organizing center (MTOC) in HEp-2 transfected cells

**Figure 7:**
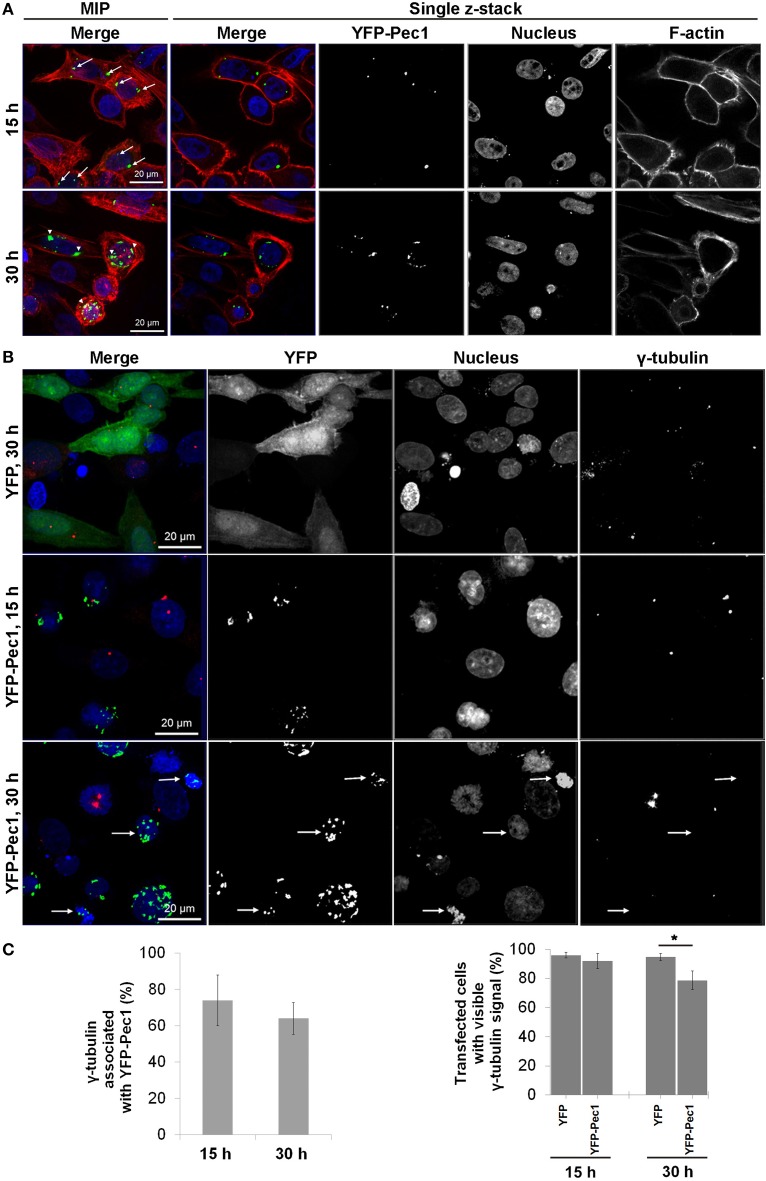
**Ectopic production and localization of YFP-Pec1 in human transfected cells. (A)** HEp-2 cells were transfected with YFP-Pec1 expressing vector for 15 h (upper panels) or 30 h (lower panels). YFP-Pec1 (green) accumulates at the nucleus poles (blue) (indicated by arrows and arrowheads in the upper and lower panels, respectively). Actin filaments (F-actin) in red. Data shown are maximum intensity projection (MIP) images or single z-sections as indicated. **(B)** Transfected HEp-2 cells expressing YFP (green) or YFP-Pec1 (green) for 15 and 30 h as indicated, were stained with anti-γ-tubulins antibody (red) to identify MTOC (arrows indicate transfected cells that lost the γ-tubulins signal). **(C)** MTOC associated with YFP-Pec1 (green) and transfected cells showed stained γ-tubulins was quantified (upper and lower graphs, respectively). The data are expressed as the means ± SEM (*n* = 3, each involving observation of 50 transfected cells). ^*^P < 0.1.

Ectopic production of YFP-Pec1 in HEp-2 cells for 15 h revealed a punctuate staining mainly with 1–4 signals per cell. Often the YFP-Pec1 dots were found to be associated with nuclei and quantification revealed that in more than 50% of transfected cells the dots were located on opposite sides of the nucleus (indicated by arrows in the upper panel of Figure 7A, MIP merge). Longer expression time (30 h) resulted in an increase in the number of dots (up to 20 signals) occasionally showing some clustering mostly associated with the nuclei (indicated by arrowheads in the lower panel of Figure 7A). In particular, the 15 h localization pattern was reminiscent of the localization of the microtubule-organizing center (MTOC). Therefore we investigated whether YFP-Pec1 is colocalizing with γ-tubulin which is a central component of the MTOC (Kollman et al., 2011). Control cells producing YFP alone showed 1–4 γ-tubulin signals per cell (Figure 7B, upper panel). After 15 and 30 h of YFP-Pec1 production, we observed γ-tubulin signals in most cells and the YFP-Pec1 producing cells showed the γ-tubulin signals directly attached to or near (1–3.2 μm) the accumulating YFP-Pec1 signals (Figure 7B, middle and lower panels). Quantification revealed that 74 and 60% of the transfected cells showed the γ-tubulin signals associated with the fusion YFP-Pec1 after 15 and 30 h of transfection, respectively (Figure 7C left). However, often cells producing the fusion protein did not show visible signals of γ-tubulin (indicated by arrows in the lower panel of Figure 7B). Again quantification revealed that cells producing the YFP-Pec1 fusion for 30 h concomitantly had lost the γ-tubulin signals in 21% [*P* < 0.1 (^*^)] of the cells in comparison to the YFP expressing cases (Figure 7C right). The results indicate that Pec1 likely is accumulating with γ-tubulin complexes and negatively influencing their number in transfected cells.

#### Tagged-Pec2 is associated with the microtubule/peroxisome/lipid droplet complex in HEp-2 transfected cells

Human cells transfected with a GFP-Pec2 expression vector showed punctuate structures of the fusion protein distributed throughout the cytoplasm (Figure S2A). In order to facilitate the analysis of Pec2 localization, we first determined the subcellular localization of GFP-Pec2 in yeast. Yeast cells producing GFP-Pec2 also showed distinct punctuate structures (1–4 dots per cell, Figures S2B–D). In order to identify possible yeast structures colocalizing with these Pec2 signals, we first visualized the yeast cytoskeleton. While the Pec2 signals did not colocalize with actin structures which appeared normal in Pec2 expressing yeast (data not shown), they strikingly associated with microtubules (MT) (Figure S2B). Further localization studies using mRFP yeast reporter strains (in which different cellular compartments are labeled with mRFP (Huh et al., 2003) revealed that GFP-Pec2 localizes with peroxisomes (Figure S2C) and lipid droplets (LD) (Figure S2D).

**Figure 8:**
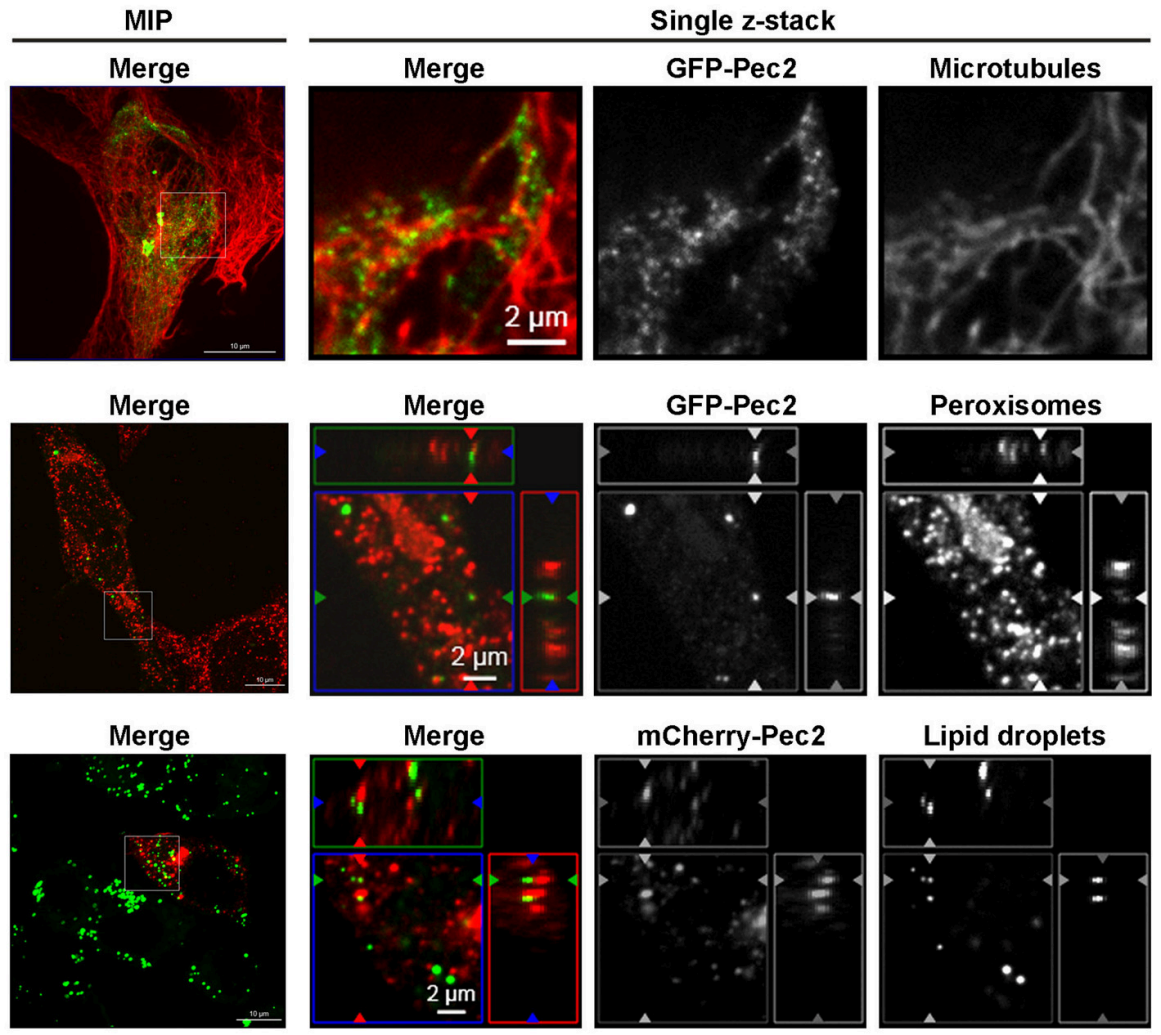
**Association of Pec2 with microtubules, peroxisomes, and lipid droplets**. HEp-2 cells were transfected with either GFP-Pec2 (green, upper, and middle panels) or mCherry-Pec2 (red, lower panels) for 18 h. Transfected cells were then fixed and labeled with either anti-alpha-tubulin (upper panels) to visualize microtubules, anti-PMp70 (middle panels) to visualize peroxisomes or Bodipy 493/503 (lower panels) to visualize lipid droplets (green). The enlarged images of single z-sections correspond to the boxes in the MIP images.

In order to test if these localization patterns of Pec2 in yeast were also conserved in human cells, we first investigated the association of the fusion protein GFP-Pec2 with MT. Fluorescence microscopy confirmed that 97.8% (± 1.67%) of the GFP-Pec2 protein signals are associated with MT structures in transfected HEp-2 cells (Figure 8). We also stained transfected HEp-2 cells for peroxisomes and LD. GFP-Pec2 producing HEp-2 cells stained with α-PMP70 antibody showed a localization of 83.3% (± 3.53%) of GFP-Pec2 signals to peroxisomes (Figure 8). Finally the analysis of HEp-2 cells producing mCherry-Pec2 stained with Bodipy showed some association of the GFP-Pec2 fusion signals 58% (± 4.37%) with lipid droplets (Figure 8). These results suggest that the LD and its associated organelles: microtubule and peroxisome (Goodman, 2008) are targets for Pec2.

## Discussion

We performed a genome-wide screen of the PA14 *P. aeruginosa* genome for bacterial proteins causing a growth inhibition phenotype in yeast. In contrast to previous studies based on selected candidates (Sisko et al., 2006; Arnoldo et al., 2008; Siggers and Lesser, 2008; Curak et al., 2009), the present work covers the whole genome and aimed at revealing unsuspected new virulence factors of the human pathogen *P. aeruginosa*. Following a 3-stage selection, 51 independent candidates were finally experimentally selected. One known virulence factor of *P. aeruginosa*, the protease LepA, was recovered in the list and therefore validates the efficiency of our screen. In addition, two known exoproteins secreted into the extracellular medium by *P. aeruginosa* were also highlighted by the screen, the non-hemolytic phospholipase C PlcN (PA14_21110) and the T1SS secreted protein AprX (PA14_48140), raising to 3 the number of secreted exoproteins recovered in our screen. However, and in contrast to LepA, PlcN, and AprX have not been demonstrated to be directly involved in *P. aeruginosa* virulence so far. Our screen however revealed that they are toxic in yeast and might therefore constitute new putative virulence factors.

Among the 51 candidates, 3 proteins were specifically selected as putative *Pseudomonas* effector candidates (Pec1, Pec2, and Pec3) and further tested under natural infection conditions. Our analysis demonstrates the significant contribution of the three Pecs to virulence in the *C. elegans* slow killing assay, while Pec1 and Pec3 also contribute to *P. aeruginosa* acute cytotoxicity toward macrophages. These results prove that our selection procedure, i.e., search for bacterial ORFs inducing growth phenotype when expressed in yeast, followed by the *in silico* sorting of hypothetical soluble proteins is efficient to select true virulence factors. Interestingly, while required for *P. aeruginosa* virulence in the worm model, Pec2 has no influence on the cytotoxicity toward macrophages, which may suggest that Pec2 does not naturally target this kind of immune cells. This is indeed the case for the H2- and H3-T6SS (Type VI secretion systems) and their cognate virulence effectors that are required for internalization of *P. aeruginosa* into epithelial cells but display no cytotoxicity against phagocytic cells (Sana et al., 2012, 2015; Jiang et al., 2014).

We are aware that the stringency of the whole candidate selection procedure followed during this screen may have led to the exclusion of additional virulence factors. Therefore, a different *in silico* selection leading to other Pecs from the 51-list could have been done using alternative criteria. Indeed, not all bacterial soluble translocated proteins necessarily inhibit yeast growth. A previous study indeed showed that only half of the 19 known *Shigella* translocated proteins tested significantly inhibit yeast growth (Slagowski et al., 2008). Thus, it would be interested in the future to test the complete initial list of 51 effector candidates.

The most potent *Pseudomonas* cytotoxin, ToxA (69 kDa) and ExoU (74 kDa) (Bleves et al., 2010) have not been recovered in our screen. This could be due to the size-limitation of the genomic fragment expressed by the library (maximum 1000 bps equivalent to 37 KDa), which does not allow the production of full length ToxA and ExoU which apparently need to be full size to be active. This is however not the case for LepA (140 kDa), PlcN (77 kDa), and AprX (41 kDa) since the production of an internal fragment is sufficient to mediate cytotoxicity in yeast. Interestingly, this internal domain does not contain the serine protease or the cell attachment motifs identified in LepA (Kida et al., 2008) and have no homology to any known functional domains. Its toxic activity is therefore carried through an unknown mechanism.

The size fragment limitation of our screen can moreover explain why none of the nine ORFs (including *exoU*) that impaired the yeast growth in a previous similar yeast phenotype screen (Arnoldo et al., 2008) was recovered in our screen. Interestingly, none of the 51 Pecs revealed in our study was recovered in Arnoldo's screen (Arnoldo et al., 2008) which was restricted to 505 pre-selected candidates, thus highlighting a novel list of effector candidates never suspected before.

Since Pec1, Pec2, and Pec3 are predicted soluble proteins, toxic when produced in yeast and contribute to *P. aeruginosa* virulence, we have postulated that they are secreted virulence factors. In this respect, we extensively tried to show their secretion in the extracellular medium by *P. aeruginosa*. Thanks to specific antibodies raised against Pec1, Pec2, and Pec3 we have tried to detect them in the supernatant of various planktonic cultures of *P. aeruginosa*. Unfortunately we were unable to detect any of the Pecs inside or outside the bacterium (data not shown). This result suggests that *pec* genes are subject to a specific regulation mechanism inactive under planktonic conditions. This is in line with the host contact-dependent induction of *pec1* reported by Chugani and Greenberg (2007). We have thus cloned and over-expressed in *P. aeruginosa* the 3 corresponding *pec* genes. Although they are properly produced (data not shown), none of the artificially overproduced Pecs were detected in the extracellular medium. While we cannot exclude that Pecs are non-secreted effectors, we favor the possibility that they require partners and/or secretion machines subject to similar regulatory process and therefore not produced under the artificial growth conditions used. We also cannot exclude that they need a host factor for stability explaining why we do not detect them in the extracellular medium. Finally, since the 3 selected Pecs are lacking signal peptides, they most likely do not use T2SS or T5SS for their secretion. Understanding the secretion pathway undertaken by the Pecs will certainly provide important information on the mode of action of those 3 *P. aeruginosa* virulence factors.

Ectopically produced Pecs in HEp-2 cells revealed specific cellular localization for Pec1 and Pec2 After 15 and 30 h of expression of YFP-Pec1 in HEp-2 cells, dot-like structures are visible and about 60% of the fusion protein locates closely adjacent to the MTOC signal. Moreover, 21% of the cells expressing the fusion protein show absence of the MTOC marker γ-tubulin. This suggests that Pec1 could target the host MTOC. HEp-2 cells expressing the tagged Pec2 showed the fusion protein as punctuate structures all over the cytosol and associated with LDs and its associated organelles including MT and peroxisomes (Goodman, 2008). As vesicle transport is mediated by the host cytoskeleton, the Pec2 producing pattern might suggest an involvement of the protein in MT-dependent transport of LD and/or peroxisomes during infection. Recruitment of MT-based activities has been reported for a number of pathogens (Radhakrishnan and Splitter, 2012). To explain why GFP-Pec reporter fusions are not toxic when produced in yeast of mammalian cells, it should be noted that expression of GFP-Pec2 from the single copy plasmid used for localization experiments resulted in a very weak or normal growth in yeast compared to the 2 μ vector used to screen the library (data not shown). In general, the localization pattern of ectopically expressed effector proteins can provide novel insights into pathogenic strategies exerted by pathogens. For example, the localization of the *Chlamydia* Lda3 protein with yeast and human lipid droplets has led to the discovery of LD consumption by *Chlamydia* (Sisko et al., 2006; Cocchiaro et al., 2008). The fact that Pec1 and Pec2 are associated with host cellular organelles might lead us to investigate the role of the new host targets which will extend our understanding of the molecular mechanisms involved in the *P. aeruginosa* infection.

### Conflict of interest statement

The authors declare that the research was conducted in the absence of any commercial or financial relationships that could be construed as a potential conflict of interest.
