# Supplementary material for: Genome-wide Screen of Pseudomonas aeruginosa in Saccharomyces cerevisiae Identifies New Virulence Factors
Source: Front Cell Infect Microbiol. 2015 Nov 16;5:81. doi: 10.3389/fcimb.2015.00081 (PMC4644809; doi:10.3389/fcimb.2015.00081)
Supplement: Supplementary file 1 [file DataSheet1.PDF]

Additional files

Additional file 1: Additional materials and methods

For expression of selected ORFs, the coding sequences of the bacterial genes were amplified from genomic DNA. Plasmids were constructed by homologous recombination in *S. cerevisiae* using the lithium acetated method (1) or by ligation followed by amplification in *E. coli*. For yeast growth phenotype studies, the coding sequences of the bacterial genes were integrated in frame C-terminal to the His_6-tag at the *EcoR*V restriction site in the empty library vector pSR1. For localization of Pec2 in yeast, the coding sequence was cut out of pSR1 using *Spe*I and *Xho*I and inserted by ligation in frame to *gfp* into the same sites in pRZ10 (*HIS3 GAL1-gfp CEN6/ARSH4*), a p413GAL1-derived plasmid containing the *gfp* coding sequence [45]. For expression in human cells, *pec1* was amplified using oligonucleotides containing the sequence of proper cutting sites (*Bam*HI and *Bgl*II). The PCR product was then cut using *Bam*HI and *Bgl*II and inserted in frame to the YFP coding sequence in the human expression plasmid pEYFP-C1 (Clontech). *pec2* and *pec3* were cloned in frame to *gfp* by homologous recombination using the lithium acetated method in pBYE (a derivative of pcDNA3.1/NT-GFP (Invitrogen), bearing a PCR-amplified *CEN6 ARSH4 TRP1* cassette integrated into the *Dra*III site to allow cloning of DNA fragments by homologous recombination in *S. cerevisiae*).

Additional file 2: Figure S1 legend. Primary screen: colony morphology screen of yeast cells expressing *P.aeruginosa* PA14 genomic library. Yeast cells were transformed and grown on SD-HIS supplemented with raffinose (upper panel). Colonies obtained were transferred by replica platting to a solid SD-HIS containing galactose medium to induce the expression of library inserts and Phloxine B to identify dead cells (lower panel). Arrows and numbers in boxes in the left panel represent examples for abnormal morphology of yeast colonies observed (non-growing (1), dead phloxine-positive (2) and smaller colonies (3)) and the corresponding colonies on the SD-HIS supplemented with raffinose plates. Lower panels are enlargement of the boxes above.

Additional file 3: Figure S2 legend. Ectopically produced GFP-Pec2 in human and yeast cells and its association with microtubules, peroxisomes and lipid droplets in yeast. (a) HEp-2 cells were transfected with GFP-Pec2 for 18h. Transfected cells were then fixed and visualized. (b) Wild type, mRFP-Pex3 and mRFP-Erg3 yeast strains (upper, middle and lower panels, respectively) were transformed with an expression vector carrying GFP-Pec2. Yeast cells expressing GFP-Pec2 were fixed and subjected for microscopical analysis. For wild type cells, microtubules were labeled with anti-alpha-tubulin. GFP-Pec2 is shown in green while microtubules, mRFP-Pex3 and mRFP-Erg3 are shown in red.

Additional file 4: Table S1 legend. Complete list of the 51 selected candidates with their corresponding PAO1 PA number, gene name and predicted function (when relevant).

Reference

Brown, A. and Tuite, M. (1998). , Yeast Gene Analysis. Academic Press, London.

Figure S1

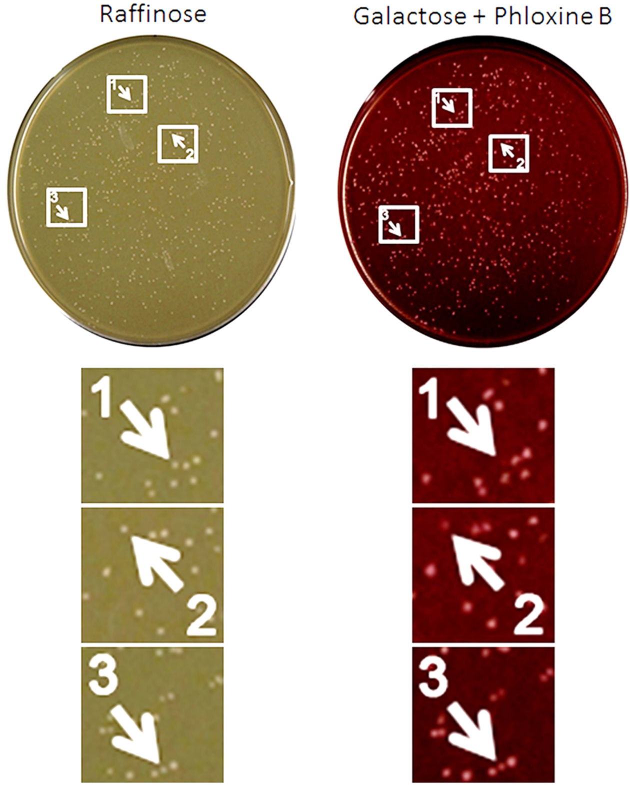

Figure S2

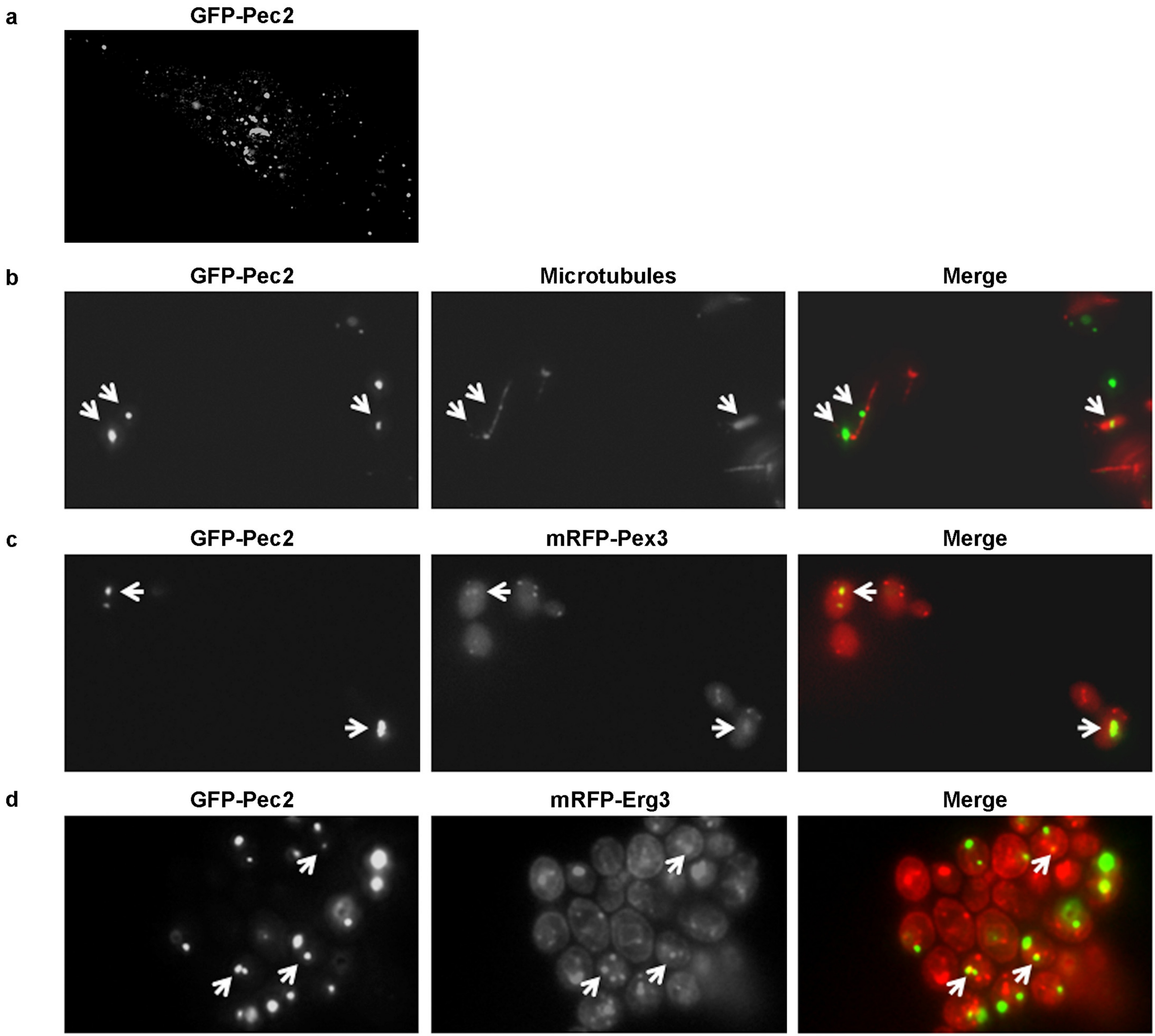
a
GFP-Pec2
b
GFP-Pec2
Microtubules
Merge
c
GFP-Pec2
mRFP-Pex3
Merge
d
GFP-Pec2
mRFP-Erg3
Merge

Table S1

PA14 number	PAO1 number	Gene name	Predicted function
PA14_68050	PA5151		Unknown
PA14_13430	PA3901	*fecA*	Fe(III) dicitrate transport protein
PA14_68640	PA5196		Putative ATP-dependent zinc protease
PA14_04520	PA0345		Unknown
PA14_36460	PA2175		Unknown
PA14_24510	PA3061	*pelD*	Extracellular polysaccharide biosynthetic pathway
PA14_70240	PA5320	*coaC*	Coenzyme A biosynthesis pathway
PA14_05540	PA0426	*mexB*	Drug transmembrane transporter
PA14_05810		*amaB*	Metabolic process
PA14_70440	PA5336	*gmk*	Metabolic process
PA14_01810	PA0147		Unknown
PA14_69670	PA5277	*lysA*	Metabolic process
PA14_08760	PA4270	*rpoB*	Transcription
PA14_47390	PA1301		Unknown
PA14_26280	PA2920		Unknown
PA14_72420	PA5487		Unknown
PA14_01830	PA0148	*add*	Deaminase activity
PA14_40150	PA1884		Unknown
PA14_29270	PA2695	*yaiW*	Unknown
PA14_57730	PA4444	*mltB1*	Peptidoglycan biosynthetic process
PA14_18430	PA3549	*algJ*	Alginate biosynthetic process
PA14_56830	PA4370	*icmP*	Unknown
PA14_27370	PA2840	*deaD*	ATP-dependent helicase activity
PA14_41530	PA1780	*nirB*	Heme biosynthetic process
PA14_12140	PA3995		Unknown
PA14_02840	PA0231	*pcaD*	Cellular catabolic process
PA14_65090	PA4929		Unknown
PA14_12820	PA3946		Unknown
PA14_43100		*rhsP2*	Self-proteolysis
PA14_71180	PA5390		Unknown
PA14_66570	PA5035	*gltB*	Glutamate biosynthetic process
PA14_68850	PA5213	*gcvP1*	Metabolic process
PA14_38140	PA2040		Unknown
PA14_02300	PA0182	*fabG*	Metabolic process
PA14_16350	PA3714		Unknown
PA14_04210	PA0322		Unknown
PA14_29880	PA2647	*nuoL*	Metabolic process
PA14_06000	PA0459	*clpA*	Metabolic process
PA14_05790	PA0443		Unknown
PA14_03090	PA0250		Unknown
PA14_68660	PA5187	*rimK*	Metabolic process
PA14_66480	PA5028		Unknown
PA14_36450	PA2176		Unknown
PA14_07450	PA0574		Unknown
PA14_03980	PA0305		Unknown
PA14_58900	PA4541	*lepA*	Type V secreted effector
PA14_21110	PA3319	*plcN*	Type II secreted exoprotein
PA14_48140	PA1243	*aprX*	Unknown (type I secreted exoprotein)
PA14_52910	PA0878	*pec1*	Unknown
PA14_03100		*pec2*	Unknown
PA14_48530	PA1221	*pec3*	AMP-binding enzyme
